# Monthly variations in aneurysmal subarachnoid hemorrhage incidence and mortality: Correlation with weather and pollution

**DOI:** 10.1371/journal.pone.0186973

**Published:** 2017-10-26

**Authors:** Myung-Hoon Han, Jinhee Kim, Kyu-Sun Choi, Choong Hyun Kim, Jae Min Kim, Jin Hwan Cheong, Hyeong-Joong Yi, Seon Heui Lee

**Affiliations:** 1 Department of Neurosurgery, Hanyang University Guri Hospital, Gyeongchun-ro, Guri, Gyonggi-do, Korea; 2 Department of Nursing, College of Medicine, Chosun University, Gwangju, Korea; 3 Department of Neurosurgery, Hanyang University Medical Center, Wangsimni-ro, Seongdong-gu, Seoul, Korea; 4 Department of Nursing Science, College of Nursing, Gachon University, Hambangmoe-ro, Yeonsu-gu, Incheon, Korea; Monash University, AUSTRALIA

## Abstract

**Background and purpose:**

Although the effect of weather and air pollution on the occurrence of subarachnoid hemorrhage (SAH) has been investigated, results have remained inconsistent. The present study aimed to determine the seasonality of aneurysmal subarachnoid hemorrhage occurrence and mortality.

**Methods:**

We used the National Inpatient Sample database to evaluate the effect of meteorological factors and air pollutants on patients with subarachnoid hemorrhage in Korea between 2011 and 2014. Monthly variations in SAH occurrence and mortality were analyzed using locally weighted scatter plot smoothing curves. Multivariate Poisson generalized linear regression models were used to evaluate potential independent meteorological and pollutant variables associated with SAH occurrence and mortality.

**Results:**

In total, 21,407 patients who underwent clip or coil treatment owing to aneurysmal SAH in Korea from January 1, 2011, to December 31, 2014, were included. The crude incidence rate of SAH in Korea was 10.5 per 100,000 people per year. An approximately 0.5% lower risk of SAH was observed per 1°C increase in mean monthly temperature (relative risk, 0.995; 95% confidence interval [CI], 0.992–0.997; p < 0.001), while an approximately 2.3% higher risk of SAH was observed per 1°C increase in mean monthly diurnal temperature.

**Conclusions:**

We showed distinct patterns of seasonal and monthly variation in the occurrence and mortality of SAH. Our findings suggest that meteorological factors may play an important role in monthly variations in the occurrence of aneurysmal SAH.

## Introduction

The influence of weather and air pollution on the occurrence of aneurysmal subarachnoid hemorrhage (SAH) has been widely investigated numerous countries; however, their results remain inconsistent. Although several researchers have reported that the incidence of SAH is lower in the summer,[1,2] additional studies have revealed no seasonal variability in the occurrence of SAH.[3–6] Various meteorological factors such as temperature, atmospheric pressure, and other weather parameters have been investigated to evaluate their possible association with SAH seasonality and mortality.[2,7–13] Several hypothesized mechanisms linking temperature, SAH occurrence, and mortality have been suggested. Colder temperature may be associated with higher SAH occurrence and mortality by increased fibrinogen levels, increased blood pressure and sympathetic nerve activity, and increased systemic infection rates.[11] In addition, the possible link between SAH occurrence/mortality and air pollution has also been investigated.[14–17] Exposure to pollutants was suggested to be linked to deterioration of vascular endothelial function, increased systemic inflammation and platelet activation, and decreased antioxidant enzyme activity.[18]

We aimed to determine the seasonality of aneurysmal SAH occurrence and mortality. In addition, we used the National Inpatient Sample (NIS) database to evaluate the effect of meteorological factors and air pollutants on SAH occurrence and mortality in Korea from 2011 to 2014.

## Methods

### Data collection

This study was approved by the institutional review board of Gachon University (IRB No. 1044396-201606-HR-043-01). Owing to the retrospective nature of the study, the need for informed consent was waived.

We collected NIS data on patients with SAH from January 1, 2011, to December 31, 2014, available by the Health Insurance Review and Assessment Service (HIRA; http://opendata.hira.or.kr) in Korea each year. Detailed information regarding the NIS and HIRA has been described elsewhere.[19,20] As each person has a unique resident registration number, duplication of patients can be avoided.[21] All patients with primary or secondary SAH diagnosis codes of I60 to I609, based on the International Classification of Diseases, tenth edition (ICD-10), were searched and extracted. Initially, we identified a total of 71,737 patients with spontaneous SAH. However, our initial search captured data from patients with prolonged hospital owing to sequelae of SAH that had occurred prior to 2011, idiopathic or non-aneurysmal SAH, and admission diagnosis code errors. To enhance the reliability of detecting monthly variation in true cases of newly developed aneurysmal SAH between 2011 and 2014, we only included patients who had undergone treatment procedures (surgical clipping or endovascular coiling), despite the potential for underestimation of the incidence rate. Therefore, we extracted data from patients with procedure codes for clipping (S4641, simple; S4642, complex) or coiling (M1661, assisted coiling; M1662, other (simple) coiling) from among the initial sample of 71,737 patients. When both clipping and coiling were performed in the same individual, the case was regarded as either clipping or coiling based on the procedure performed at the earliest date. We finally included 21,407 patients, who were followed up until December 31, 2015, to obtain data regarding SAH mortality. Sex, age, demographic distribution of hospital admissions, dates of hospital admission and discharge, and other variables were extracted for all patients. We defined the day of onset of SAH based on the admission date of the patient. The number of SAHs occurring each month was also recorded.

### Mortality assessment

The 21,407 selected study patients were followed up until December 31, 2015, using data from the NIS database. Since the HIRA does not provide mortality information,[19] the mortality date was defined as the date on which the last discharge code was recorded, when satisfying the following criteria: (1) visited the outpatient department ≦2 times (in case of death certificate) with no medication prescribed, and no further admission within 3 months post-discharge, and (2) did not use medical facilities for any purpose in Korea until December 31, 2015, after 3 months post-discharge. All Koreans are beneficiaries of the Korean National Health Insurance System, thus HIRA database enabling to trace records of nation-wide inpatient and outpatient data.[22] Therefore, we could identified all records of SAH patients visiting any medical facilities including primary hospitals, private clinics, public health centers, and pharmacies in South Korea.[23] In treated SAH patients, all patients (even patients with modified Rankin scale 0 at discharge) were to be followed up by a physician and prescribed medication in an outpatient department (at the hospital where patient was treated or any other hospitals in Korea), especially during the early period after discharge. Most patients in a vegetative state are transferred to a rehabilitation hospital or nursing facility. Even if few vegetative patients may transfer to their home, the guardians may need to use medical facilities to administer prescribed medications. When the vegetative patient is found dead by the guardian at home, the dead body must be carried to the emergency room for cadaver examination, and subsequently, the discharge code should be recorded in the emergency room. Therefore, we assumed the date of death to be the day of discharge after clip or coil treatment when these conditions are met. We present the detailed SAH admission and mortality data based on each month, classified by sex and age group (S1 Data) We also performed sensitivity analysis to evaluate the suitability of our criteria with a cut-off value of 3 months. No significant differences in mortality were observed when each month (2–5) was set as the cut-off value (S1 Table).

### Meteorological and air pollution data

Meteorological data including monthly measures of mean temperature, average diurnal temperature range, and insolation from 2011 to 2014 in Korea were obtained from the Meteorological Administration of South Korea (http://www.kma.go.kr/eng/). These data were collected and analyzed from 73 observation stations located throughout South Korea.[24]

Air pollutant data included average monthly measures of particulate matter with an aerodynamic diameter < 10 μm (PM_10_), nitrogen dioxide (NO_2_), and sulfur dioxide (SO_2_) in Korea for the duration of the study period. These data were collected from Air Korea, which was established in 2002 by the Ministry of Environment of South Korea (www.airkorea.or.kr/eng/). The comprehensive air pollution data were collected and analyzed from 251 sites in 79 cities.[25]

### Season

Seasons were defined as winter (December through February), spring (March through May), summer (June through August), and autumn (September through November).

### Statistical methods

The crude SAH incidence rate was estimated as the average number of SAHs occurring in the study period per 100,000 people per year. Discrete variables are expressed as percentages; continuous variables, the mean ± standard deviation or median with interquartile range (IQR).

Descriptive statistics were used to determine the median and IQR of monthly meteorological and air pollution measures. The relationships among meteorological and air pollution factors were evaluated using the Pearson correlation test.

We used a locally weighted scatter plot smoothing (LOWESS) curve with a 95% confidence interval (CI) to graphically represent the total monthly variation in the occurrence/mortality of aneurysmal SAH, which was then stratified in accordance with each year. Student’s *t*-test was used to identify seasonal variations in SAH occurrence and mortality. Linear regression lines for the LOWESS curve were used to identify the association among temperature, diurnal temperature range, and SAH occurrence. SAH occurrence was natural log-transformed to normalize distributions.

Univariate and multivariate Poisson generalized linear regression models were used to evaluate potential independent meteorological and pollutant variables associated with SAH occurrence and mortality, using a log-linkage function offset by the log of the population in each year from 2011 to 2014. The population of Korea between 2011 and 2014 was investigated based on the Korean population census (http://rcps.egov.go.kr).

We used the ggplot function in R to visualize the map of South Korea using “ggplot2” and “Kormaps” packages (e.g. ggplot (kormap1,aes (x = long, y = lat,group = group, fill = id)) +geom_polygon (colour = "black")+…). We created a map of South Korea in Excel to display geographic distribution of average temperature, diurnal temperature range, and PM_10_ concentrations using a gradient spectrum of two colors. The scatterplot with LOWESS curve and linear line were produced by “moonBook”, “ggthemes”, and “ggplot2” packages (e.g. ggplot (data = RRR, aes (x = factor(Month), y = Total, group = 1))+ stat_smooth (colour = "#2E9FDF", fill = "gray75",size = 1.1)+geom_point (fill = "#2E9FDF",size = 2,shape = 21) +… and ggplot (data = RRR, aes (x = Temperature, y = log_SAH,group = 1))+ stat_smooth (method = lm, size = 1.1, aes (colour = "Linear"), se = FALSE)+stat_smooth (size = 1.1, aes (colour = "LOWESS"), se = FALSE)+…). All statistical analyses were performed using R version 3.3.2.

## Results

### Demographic distribution of SAH occurrence and regional heterogeneity of the meteorological factors and pollutants in Korea

**Fig 1:**
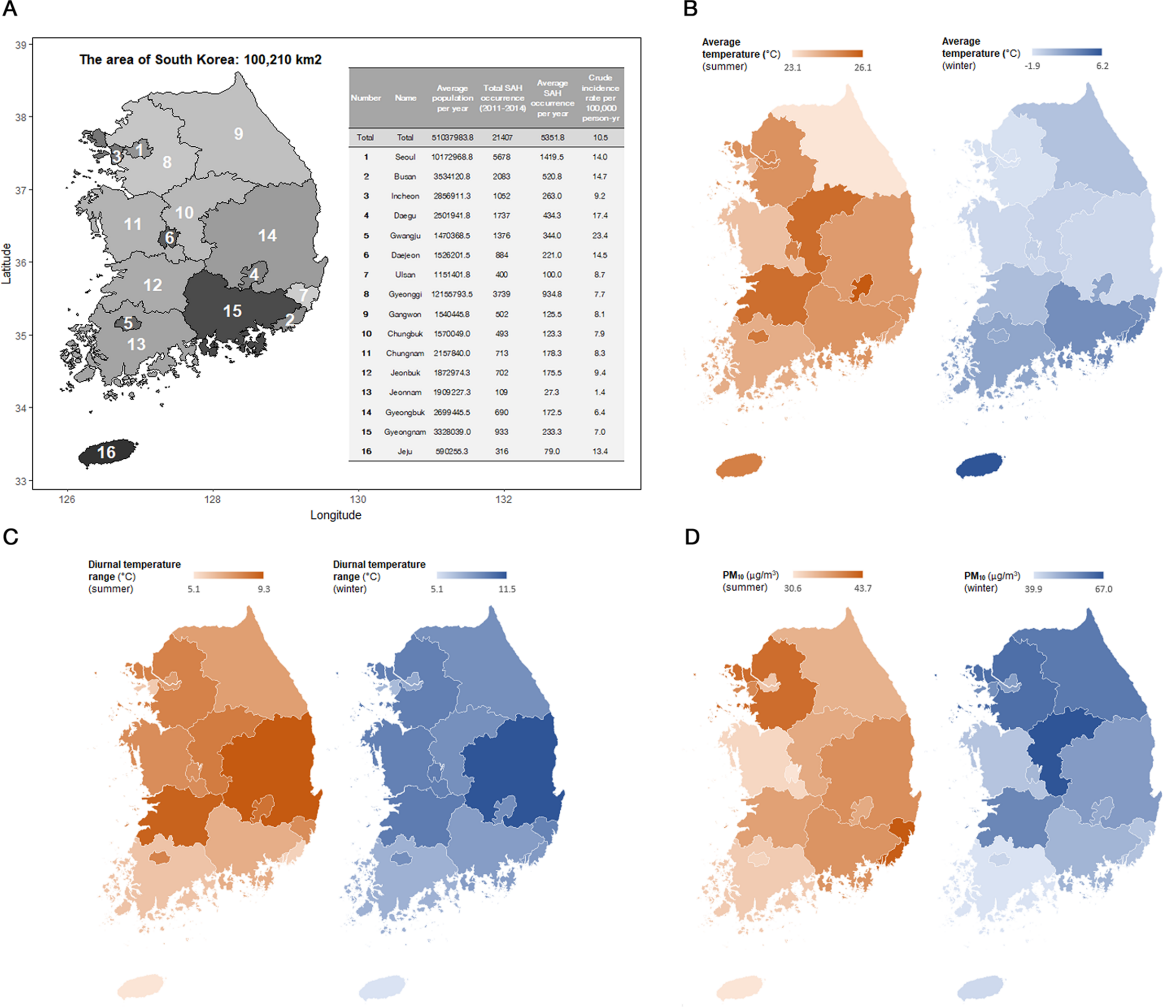
**Demographic distribution of patients who underwent procedures for aneurysmal subarachnoid hemorrhage and regional heterogeneity of the meteorological factors and pollutants in South Korea between 2011 and 2014:** (A) demographic distribution of subarachnoid hemorrhage occurrence (B) regional average temperature based on summer and winter seasons; (C) regional diurnal temperature range based on summer and winter seasons; (D) regional particulate matter with an aerodynamic diameter < 10 μm concentrations based on summer and winter seasons.

During the study period, the average population of Korea was 51,037,983.8 (Table 1 and Fig 1).

**Table 1:** Characteristics of the average population and patients with spontaneous aneurysmal subarachnoid hemorrhage in Korea between 2011 and 2014.

Characteristics	Average population of Korea between 2011 and 2014
Overall, mean	51,037,983.8
Men, mean (%)	25,542,156.5 (50.1)
Women, mean (%)	25,495,827.3 (50.0)
< 65 years, mean (%)	44,924,827.5 (88.0)
≥ 65 years, mean (%)	6,113,156.3 (12.0)
	Patients receiving treatment due to spontaneous aneurysmal SAH in Korea
Overall, n	21,407
Crude incidence rate per 100,000 person-year	10.5
Sex	
Men, n (%)	7,679 (35.9)
Women, n (%)	13,728 (64.1)
Age, mean ± SD (median)	56.3 ± 12.9 (55)
< 65 years, n (%)	15,470 (72.3)
≥ 65 years, n (%)	5,937 (27.7)
Treatment	
Clip, n (%)	11,485 (53.7)
Coil, n (%)	9,922 (46.3)
Length of stay, median (IQR)	20 (12–29)
Charlson co-morbidity Index, mean ± SD (median)	2.3 ± 1.5 (2)
In hospital mortality, n (%)	1,926 (9.0)
Mortality rate, %	9.0
Medical charges, median (IQR), ₩	13,833,740 (10,564,160 to 18,790,240)
Number of SAHs per year	
2011, n (%)	5,471 (25.6)
2012, n (%)	5,255 (24.5)
2013, n (%)	5,307 (24.8)
2014, n (%)	5,374 (25.1)

SAH, subarachnoid hemorrhage; SD, standard deviation; IQR, interquartile range

In total, 21,407 patients underwent surgical clipping or endovascular coiling owing to aneurysmal SAH between 2011 and 2014. The crude incidence rate of SAH in Korea was 10.5 per 100,000 people per year. The incidence rate was highest in Gwangju (23.4) and lowest in Jeonnam (1.4) (Fig 1A). One possible explanation for this difference is that the Regional Emergency Medical Center is in Gwangju, which is located inside Jeonnam. In addition, Jeonnam inhabitants preference for the hospital in Gwangju also affected the difference, despite an emergency situation. Fig 1B, 1C and 1D show regional differences of average temperature, diurnal temperature range, and PM_10_ in South Korea between 2011 and 2014 based on summer and winter seasons. The average temperature varied by about 3°C ~ 8°C and 4°C ~ 6°C variation of diurnal temperature range throughout nation from 2011 to 2014 in summer and winter. PM_10_ ranged from approximately 13 μg/m^3^ to 27 μg/m^3^. We also present detailed regional measures of the meteorological factors and air pollutants in South Korea each year classified by summer and winter seasons (S2 Data).

### Characteristics of the study population

The average age of patients at SAH occurrence was 56.3 years, and 64.1% of patients were women. Surgical clipping was performed in 11,485 (53.7%) patients. The median length of admission was 20 days, and the mortality rate was 9.0%. Patient characteristics are further detailed in Table 1.

### Monthly measures of meteorological factors and pollutants

Table 2 shows the monthly distribution of meteorological parameters and air pollutants with median and IQR values from 2011 to 2014. The pairwise Pearson correlation coefficients among meteorological factors and pollutants were estimated (S2 Table). Temperature exhibited a somewhat significant correlation with air pollution variables, with the strongest negative correlation observed for NO2 (r = - 0.758). Diurnal temperature range was positively correlated with insolation (r = 0.787).

**Table 2:** Monthly subarachnoid hemorrhage occurrence and average monthly meteorological factors and air pollutants in Korea between 2011 and 2014.

		Meteorological factors			Pollutants		
Month	SAH occurrence, n (%)	Temperature (°C), median (IQR)	Diurnal temperature range (°C), median (IQR)	Insolation (hour), median (IQR)	PM_10_ (μg/m^3^), median (IQR)	NO_2_ (ppb), median (IQR)	SO_2_ (ppb), median (IQR)
Jan	1954 (9.1)	- 1.7 (- 3.8 to 0.1)	10.0 (9.6 to 10.8)	196.3 (183.0 to 214.4)	57.3 (44.9 to 61.1)	26.6 (23.4 to 28.7)	7.5 (6.3 to 7.6)
Feb	1735 (8.1)	1.3 (- 0.4 to 2.4)	10.2 (10.0 to 11.0)	173.0 (150.4 to 213.5)	51.4 (48.3 to 64.1)	24.6 (23.6 to 28.7)	6.4 (5.9 to 6.7)
Mar	1960 (9.2)	6.2 (4.9 to 7.4)	11.3 (10.1 to 12.8)	226.6 (187.9 to 246.5)	58.5 (51.5 to 61.8)	22.0 (20.9 to 23.3)	5.5 (5.3 to 5.8)
Apr	1815 (8.5)	11.2 (10.5 to 12.9)	12.2 (12.1 to 12.2)	213.2 (212.5 to 217.2)	53.6 (51.5 to 56.4)	22.2 (20.2 to 36.2)	4.9 (4.8 to 5.2)
May	1779 (8.3)	18.1 (17.4 to 18.4)	11.8 (10.9 to 12.7)	236.2 (191.0 to 278.5)	62.3 (56.8 to 71.1)	20.3 (19.4 to 20.6)	5.2 (4.8 to 5.3)
Jun	1659 (7.7)	22.0 (21.9 to 22.5)	9.2 (9.0 to 9.5)	179.6 (173.1 to 188.0)	44.2 (39.9 to 46.7)	16.7 (15.1 to 17.9)	4.6 (4.3 to 5.0)
Jul	1612 (7.5)	25.3 (25.1 to 26.1)	7.4 (6.9 to 8.2)	157.5 (123.4 to 177.1)	33.9 (31.8 to 37.7)	14.4 (13.3 to 14.8)	4.2 (4.1 to 7.1)
Aug	1628 (7.6)	25.8 (24.2 to 27.1)	7.3 (6.9 to 8.6)	142.0 (114.1 to 223.7)	30.1 (26.8 to 36.0)	14.2 (12.7 to 14.6)	3.8 (3.6 to 4.3)
Sep	1642 (7.7)	21.0 (20.4 to 21.2)	9.5 (9.1 to 10.0)	184.9 (180.2 to 198.1)	32.6 (32.3 to 32.9)	16.7 (16.2 to 16.8)	4.0 (3.8 to 4.7)
Oct	1915 (8.9)	14.6 (13.9 to 15.3)	11.7 (11.5 to 12.1)	224.9 (206.8 to 233.3)	38.1 (35.5 to 43.7)	21.7 (20.1 to 23.4)	4.1 (3.9 to 4.2)
Nov	1919 (9.0)	8.0 (6.7 to 10.5)	10.0 (9.3 to 10.4)	177.8 (140.7 to 190.2)	44.2 (43.8 to 46.3)	23.4 (22.5 to 25.2)	4.9 (4.8 to 5.2)
Dec	1789 (8.4)	0.1 (- 1.4 to 1.3)	9.2 (9.0 to 9.3)	179.3 (173.4 to 188.5)	43.5 (41.0 to 49.4)	24.5 (23.4 to 25.9)	5.9 (5.7 to 6.2)

SAH, subarachnoid hemorrhage; IQR, interquartile range; PM_10_, particulate matter less than 10 mm in aerodynamic diameter; NO_2_, nitrogen dioxide; SO_2_, sulfur dioxide

### Monthly variations of SAH occurrence and mortality

**Fig 2:**
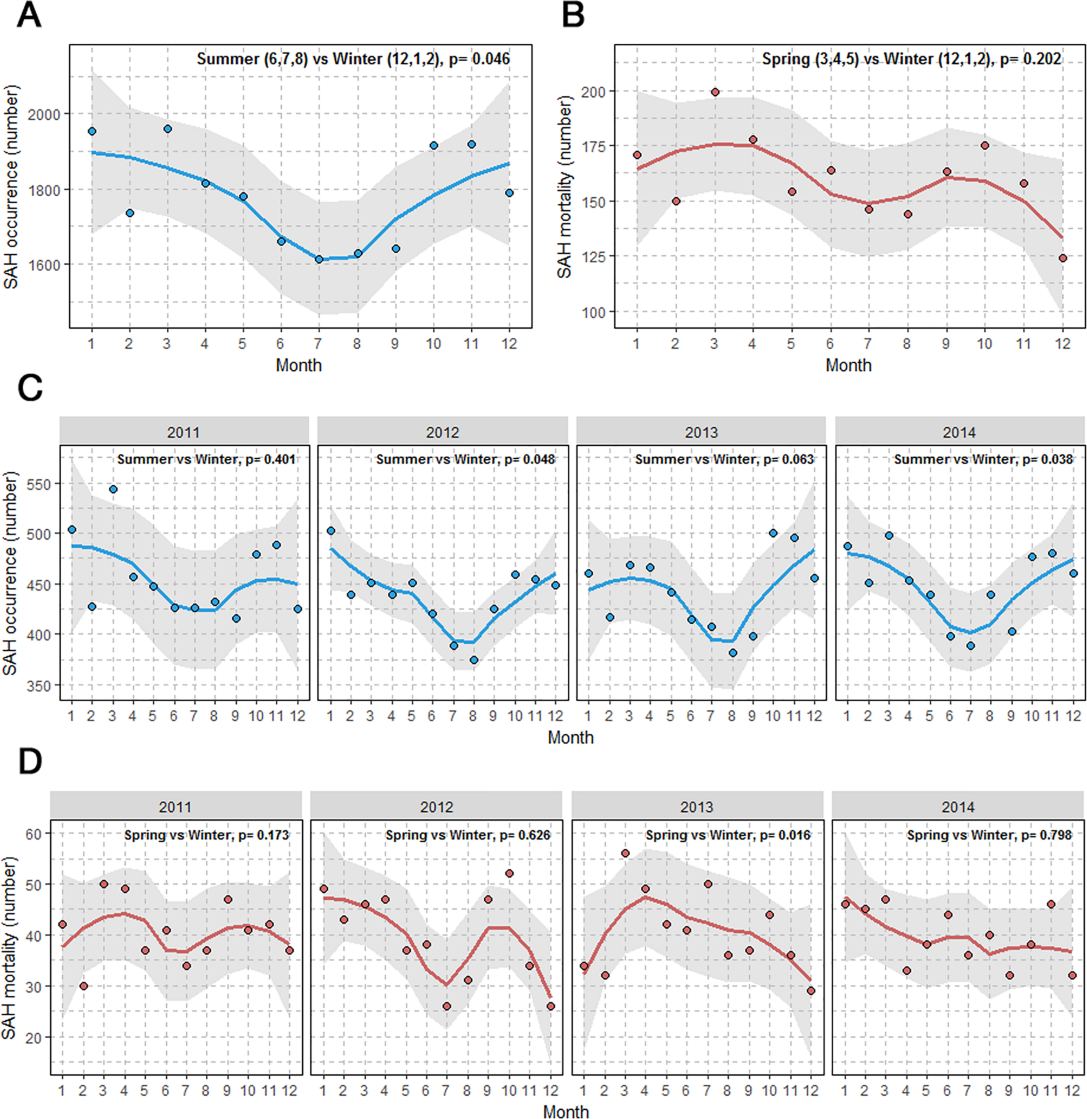
LOWESS regression line with 95% confidence interval showing the pattern of monthly SAH occurrence and mortality (overall and stratified by year).

SAH occurrence was highest in March (n = 1,960) and lowest in July (n = 1,612) (Table 2). The overall monthly variation in SAH exhibited a V-shaped pattern, with a tendency to decrease from January to summer and a tendency to increase from summer to December, with four peaks in January, March, October, and November (Fig 2A).

SAH occurrence was lower in the summer than in the winter each year (Fig 2C). This pattern was maintained when patients were divided into sex and age groups (S1A and S1B Fig). A significant difference in SAH occurrence between summer and winter was observed in women (p = 0.038), not in men (p = 0.104).

Mortality was highest in March (n = 199) and lowest in December (n = 124) (Fig 2B). Variations in mortality exhibited a pattern similar to those for SAH occurrence from January until early autumn. However, mortality tended to decrease at the end of the year, despite relatively higher SAH occurrence. Relatively irregular patterns of mortality were observed each year from 2011 to 2014 (Fig 2D). Monthly variations in mortality according to sex and age are depicted in Fig C and D of the S1 Fig.

### Combined effect of monthly mean temperature and diurnal temperature range on SAH occurrence

**Fig 3:**
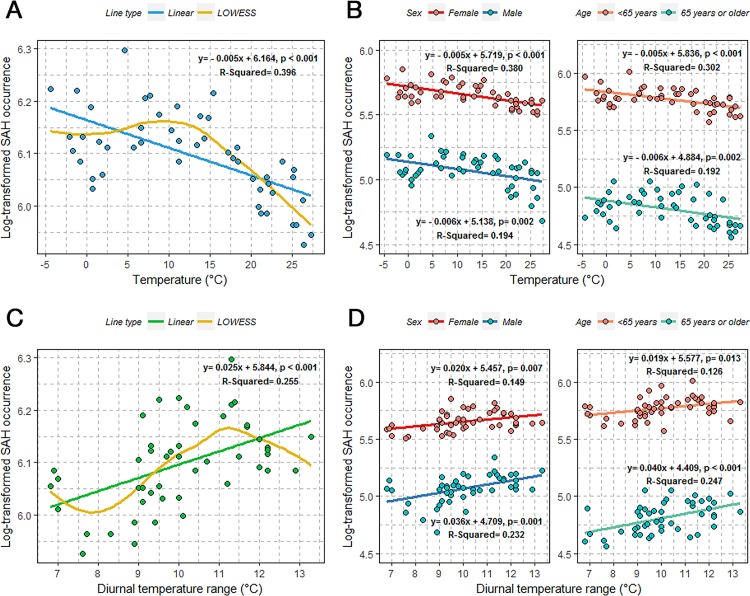
Linear and LOWESS regression curve of natural log-transformed subarachnoid hemorrhage occurrence based on temperature and diurnal temperature range, overall, and stratified according to gender and age.

The lower incidence of SAH in the summer may be somewhat explained by the effect of temperature on SAH. We observed an approximate 0.5% decrease in SAH occurrence per 1°C increase in ambient temperature, with about 40% explanatory power in the log-transformed linear regression analysis (β = - 0.005; p < 0.001; R^2^ = 0.396). A continuous downward slope was also noted for temperatures above approximately 10–15°C (Fig 3A).

This statistical significance was maintained when we divided patients into sex and age groups (Fig 3B). The results of the univariate and multivariate linear regression analyses of log-transformed SAH occurrence based on meteorological and air pollution parameters are presented in S3 Table. We observed that temperature was associated with SAH occurrence (RR, 0.995; 95% CI, 0.992–0.997; p < 0.001 per 1°C increment) in the multivariate Poisson regression after adjusting for all meteorological factors and pollutants (Table 3). This significant relationship was also observed for women, younger patients (< 65 years), and older patients (≧ 65 years) in the multivariate analysis (S4 Table).

**Table 3:** Relative risk of subarachnoid hemorrhage occurrence and mortality based on meteorological factors and air pollutants.

	SAH occurrence			
	Univariate Poisson regression		Multivariate Poisson regression	
Variable	RR (95% CI)	p	RR (95% CI)	p
Meteorological factors (per 1 unit increase)				
Temperature	0.995 (0.993 to 0.996)	< 0.001	0.995 (0.992 to 0.997)	< 0.001
Diurnal temperature range	1.025 (1.016 to 1.033)	< 0.001	1.023 (1.003 to 1.044)	0.025
Insolation	1.001 (1.000 to 1.001)	0.001	1.000 (0.999 to 1.000)	0.350
Pollutants (per 1 unit increase)				
PM_10_	1.003 (1.002 to 1.004)	< 0.001	1.000 (0.998 to 1.002)	0.811
NO_2_	1.008 (1.006 to 1.011)	< 0.001	0.999 (0.994 to 1.004)	0.672
SO_2_	1.025 (1.012 to 1.037)	< 0.001	0.995 (0.975 to 1.015)	0.616
	SAH mortality			
Variable	RR (95% CI)	p	RR (95% CI)	p
Meteorological factors (per 1 unit increase)				
Temperature	0.999 (0.994 to 1.004)	0.678	1.000 (0.991 to 1.008)	0.909
Diurnal temperature range	1.042 (1.013 to 1.071)	0.004	1.063 (0.994 to 1.137)	0.073
Insolation	1.001 (1.000 to 1.003)	0.032	0.999 (0.997 to 1.002)	0.578
Pollutants (per 1 unit increase)				
PM_10_	1.003 (0.999 to 1.007)	0.128	1.000 (0.993 to 1.007)	0.944
NO_2_	1.004 (0.995 to 1.012)	0.372	0.995 (0.979 to 1.011)	0.552
SO_2_	0.991 (0.952 to 1.033)	0.678	0.991 (0.927 to 1.061)	0.802

SAH, subarachnoid hemorrhage; RR, relative risk; CI, confidence interval; PM_10_, particulate matter less than 10 mm in aerodynamic diameter; NO_2_, nitrogen dioxide; SO_2_, sulfur dioxide

Higher SAH occurrence was also observed with higher diurnal temperature changes during the change of seasons in March, October, and November. Diurnal temperature range exhibited a positive association with SAH occurrence, with approximately 26% explanatory power (β = 0.025; p < 0.001; R^2^ = 0.255). An upward slope was also observed between 8–11°C in the LOWESS curve (Fig 3C). This association persisted when patients were divided into sex and age groups (Fig 3D). Multivariate Poisson regression revealed an approximately 2.3% increased risk of SAH occurrence per 1°C increase in average monthly diurnal temperature range (RR, 1.023; 95% CI, 1.003–1.044; p = 0.025) (Table 3). With regard to SAH mortality, diurnal temperature range and insolation showed significant association in the univariate analysis (RR, 1.042; 95% CI, 1.013–1.071; p = 0.004; RR, 1.001; 95% CI, 1.000–1.003; p = 0.032, respectively). However, there were no associations between meteorological and air pollution factors and SAH mortality in the multivariate analysis.

Monthly ambient temperature was significantly associated with SAH occurrence among women and in both age groups, while diurnal temperature range exhibited a positive association with SAH occurrence in men (RR, 1.049; 95% CI, 1.014–1.085; p = 0.006), after adjusting for all meteorological factors and pollutants (S4 Table).

## Discussion

In the present study, we found that both mean monthly and diurnal temperature range affect SAH occurrence. Lower mean temperatures were significantly correlated with higher aneurysmal SAH occurrence. In addition, higher diurnal temperature range was positively associated with SAH, this association being significantly more prominent in men than in women. In-hospital mortality was highest in March and tended to decrease at the end of the year, despite an increase in the incidence of SAH at this time. Mortality exhibited no association with either meteorological factors and pollutants in the multivariate analysis.

Previous studies have supported our finding that the incidence of SAH is lower in the summer than in other seasons.[26–28] A recent meta-analysis also reported that aneurysmal SAH occurrence was low in summer than in winter months, peaking in January.[29] Previous studies have reported inconsistent findings regarding the association between ambient temperature and SAH occurrence. In the present study, we also observed low linear correlation between SAH occurrence and lower ambient temperature (below around 10–15°C), despite an overall significant negative correlation between SAH occurrence and temperature. Furthermore, we observed a significant correlation between SAH and mean monthly diurnal temperature range. Gill et al. reported that colder temperatures and temperature fluctuations from cold to warm to cold during fall and spring are associated with increased incidence of aneurysmal SAH.[11] Therefore, we hypothesized that cold temperature may independently affect SAH occurrence and greater fluctuations in diurnal temperature in conjunction with relatively lower ambient temperatures may synergistically affect SAH occurrence.

Cold temperatures are known to increase blood pressure.[30–33] Blood pressure and intra-arterial pressure are directly related, as increases in blood pressure produce increases in transmural pressure, which may trigger aneurysm rupture.[34] Colder temperatures can dramatically increase blood pressure in unacclimatized individuals transitioning from warm to cold temperatures.[11] We speculate that greater temperature fluctuation in environments with relatively low mean temperatures during seasonal variation may affect blood pressure variability, which may lead to an increased risk of aneurysm rupture. One study reported that changes in diurnal temperature were associated with SAH occurrence in Japanese men.[13] An additional study revealed that peak levels of SAH incidence occurred in winter for women and in fall/spring in men.[12] Our findings also indicate that diurnal temperature range is associated with SAH occurrence in men, although the mechanism underlying this association remains unknown. Anderson et al. reported that moderate-to-extreme exertion in the 2 hours before SAH was associated with a three-fold increase in SAH risk.[35] Men are more likely to engage in strenuous outdoor activities or work than women, especially in the spring and autumn.[36] Hence, SAH incidence may be higher in months with relatively greater diurnal temperature ranges among men in the present study. A previous study also described the association of significant climate change before occurrence of aneurysmal ruptured SAH in men compared to women.[37]

Several studies have reported a possible correlation between SAH occurrence and atmospheric pressure, humidity, or insolation.[7–10,13,38,39] In the present study, we observed strong correlation between the incidence of SAH and temperature, atmospheric pressure, and precipitation (S5 Table). According to Gay-Lussac's Law (k = pressure/temperature; k = constant), atmospheric pressure is dependent on air temperature. In addition, about 60% of total annual precipitation in Korea occurs during the summer (June to August). Therefore, we speculate that cases of SAH that seem to be associated with atmospheric pressure and humidity may instead be primarily attributable to changes in temperature. Several studies have also reported a possible association between hemorrhagic stroke and air pollution.[14,16,17] However, our study revealed no correlation between SAH and pollutants after adjusting for meteorological variables. However, this finding seems to confirm the short-term effects of pollutants in a confined area under a similar environment.

No significant association was observed between in-hospital SAH mortality and any average monthly meteorological factors or pollutant. Our findings indicate that mortality was highest in March, gradually decreasing from March to December with some fluctuations, in accordance with the pattern of SAH occurrence. Several studies have documented the “July effect”: a transient increase in adverse SAH outcomes during July,[40,41] probably owing to the July influx of novice interns and new medical residents with minimal clinical experience. In Korea, the influx of new medical personnel begins in March. Therefore, this factor may influence SAH mortality rate because life-threatening complications of SAH such as vasospasm, re-bleeding, and hydrocephalus are quite common. A previous nationwide Danish study also indicated that there was no seasonal variation in a 30-day SAH case fatality.[1] However, there are suggestions regarding various human mechanisms such as increased fibrinogen levels, blood pressure and sympathetic nerve activity, systemic infection rates, deterioration of vascular endothelial function, and decreased antioxidant enzyme activity, according to the environmental changes. Although our study showed no correlation between in-hospital SAH mortality and meteorological factors or pollutants, we think that these possible mechanisms that can be associated with environmental changes may also possibly play a role in case-fatality, especially among moderately to severely ill patients.

With consideration of all findings and hypotheses of the study, we think that exposure to abrupt or chronic cold temperature may affect risk of aneurysm rupture in patients with unruptured intracranial aneurysms. Therefore, precaution may be helpful for prevention of SAH in patients with unruptured aneurysms. In addition, previous studies indicated that higher body temperatures may have a larger benefit for treatment in patients with stroke.[42,43] Our findings may be extensions to the clinical management for patients with stroke considering control of body temperature with further research needed.

Our study has the following limitations. First, we could not include all patients with SAH owing to the inherent limitations of using the NIS database. However, random selection of patients with SAH who had undergone clipping or coiling procedures did not influence monthly variations in SAH. In addition, we think that this inevitable selection due to using NIS database naturally excludes milder cases and non-aneurysmal spontaneous SAH which account for between 10 and 20% of all spontaneous SAH.[44] Second, regional weather and environmental heterogeneity in Korea may lead to somewhat biased results. This environmental heterogeneity may be inevitable in nationwide studies. However, we think that the narrow range of latitude (33–38°) and relatively small area of the nation may have relatively little effect on regional temperature variations.[24,45] Third, this study included only the Korean population; hence, the generalizability of these findings is limited. Fourth, inevitable bias may be present when defining death, due to the inherent nature of using the NIS database. We previously reported several studies having similar death definitions to the present study.[22,46,47] Finally, since we used average monthly weather and pollutant data, this study cannot evaluate the short-term effects of daily variations in meteorological and air pollution levels on SAH occurrence. However, long-term studies may provide further information on the cumulative effects of chronic exposure to weather and air pollution.[48] Previous studies indicated that daily variations in temperature may have significant effects on increasing the risk of SAH.[10,11]

## Conclusions

Despite these limitations, we observed a trend toward lower SAH occurrence in the summer and higher SAH occurrence in January and during the change of seasons. These trends were significantly associated with the mean monthly temperature and diurnal temperature range. However, we observed no correlation between SAH mortality and meteorological/air pollution parameters. Our findings suggest that meteorological factors may play an important role in monthly variations in the occurrence of aneurysmal SAH, although aneurysm rupture itself is likely to be multifactorial.

## Supporting information

S1 Data The detailed SAH admission and mortality data based on each month, classified by sex and age group. (XLSX)

S2 Data Regional measures of the meteorological factors and air pollutants in South Korea each year classified by summer and winter seasons. (XLSX)

S1 Fig Scatterplot with LOWESS regression line (95% confidence interval) showing monthly SAH occurrence and mortality patterns according to gender and age group. (TIF)

S1 Table Sensitivity analysis of cut-off value for mortality. (DOCX)

S2 Table Pearson correlation coefficients among weather variables and pollutants. (DOCX)

S3 Table Univariable and multivariable linear regression of log-transformed SAH occurrence based on meteorological and air pollution parameters. (DOCX)

S4 Table Multivariate Poisson regression analysis of SAH occurrence according to sex and age group, based on meteorological factors and air pollutants. (DOCX)

S5 Table Pearson correlation coefficients between average monthly temperature and atmospheric pressure and precipitation. (DOCX)
