# Supplementary figures and images for: Monthly variations in aneurysmal subarachnoid hemorrhage incidence and mortality: Correlation with weather and pollution
Source: PLoS One. 2017 Oct 26;12(10):e0186973. doi: 10.1371/journal.pone.0186973 (PMC5658131; doi:10.1371/journal.pone.0186973)

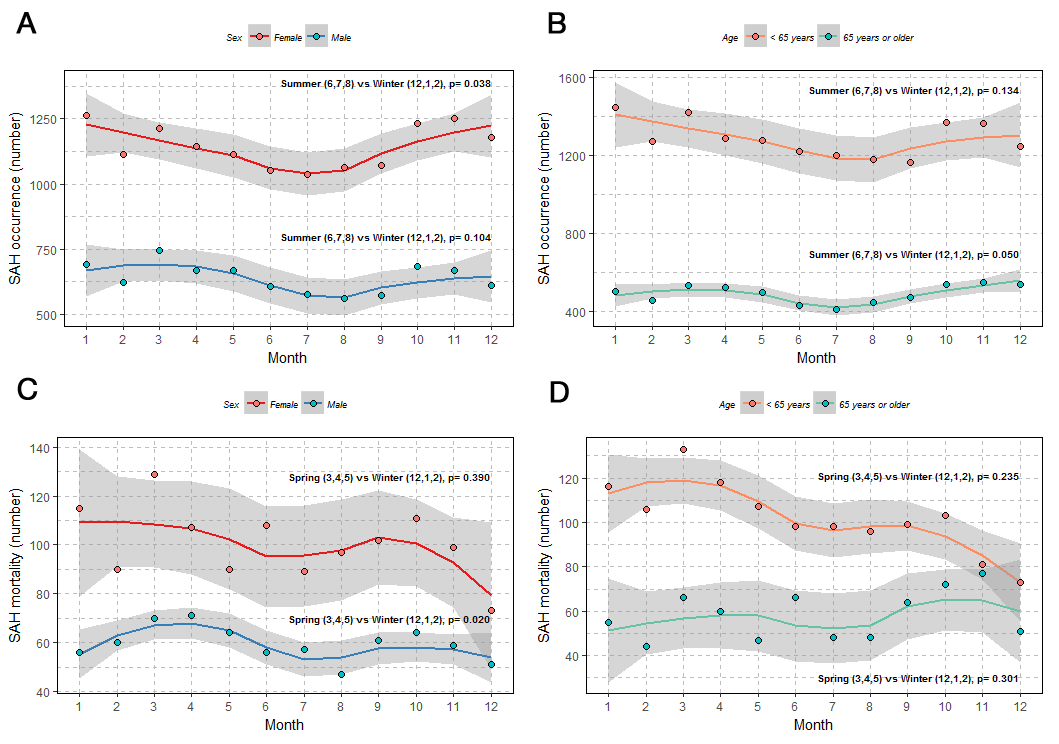

Supplement: S1 Fig — (TIF) [file pone.0186973.s003.tif]
