# Supplementary material for: Monthly variations in aneurysmal subarachnoid hemorrhage incidence and mortality: Correlation with weather and pollution
Source: PLoS One. 2017 Oct 26;12(10):e0186973. doi: 10.1371/journal.pone.0186973 (PMC5658131; doi:10.1371/journal.pone.0186973)
Supplement: S1 Table — (DOCX) [file pone.0186973.s004.docx]

|  | N | % |
| --- | --- | --- |
| Total number of patients with SAH | 21,407 | 100 |
| In-hospital mortality | | |
| Cut-off value |  |  |
| Two months | 1,925 | 9.0 |
| Three months | 1,926 | 9.0 |
| Four months | 1,926 | 9.0 |
| Five months | 1,926 | 9.0 |

SAH, subarachnoid hemorrhage
