# Supplementary material for: Monthly variations in aneurysmal subarachnoid hemorrhage incidence and mortality: Correlation with weather and pollution
Source: PLoS One. 2017 Oct 26;12(10):e0186973. doi: 10.1371/journal.pone.0186973 (PMC5658131; doi:10.1371/journal.pone.0186973)
Supplement: S2 Table — (DOCX) [file pone.0186973.s005.docx]

|  | Temperature (°C) | Diurnal temperature range (°C) | Insolation (hour) | PM_10_ (μg/m^3^) | NO_2_ (ppb) | SO_2_ (ppb) |
| --- | --- | --- | --- | --- | --- | --- |
| Temperature (°C) | 1 | - 0.369** | - 0.170 | - 0.515** | - 0.758** | - 0.672** |
| Diurnal temperature range (°C) |  | 1 | 0.787** | 0.666** | 0.552** | 0.113 |
| Insolation (hour) |  |  | 1 | 0.433** | 0.220 | - 0.007 |
| PM_10_ (μg/m^3^) |  |  |  | 1 | 0.616** | 0.520** |
| NO_2_ (ppb) |  |  |  |  | 1 | 0.518** |
| SO_2_ (ppb) |  |  |  |  |  | 1 |

PM10, particulate matter less than 10 mm in aerodynamic diameter; NO2, nitrogen dioxide; SO_2_, sulfur dioxide

**p <0.001

*p <0.05
