# Supplementary material for: Monthly variations in aneurysmal subarachnoid hemorrhage incidence and mortality: Correlation with weather and pollution
Source: PLoS One. 2017 Oct 26;12(10):e0186973. doi: 10.1371/journal.pone.0186973 (PMC5658131; doi:10.1371/journal.pone.0186973)
Supplement: S3 Table — (DOCX) [file pone.0186973.s006.docx]

|  | Univariable analysis | |  | Multivariable analysis | |
| --- | --- | --- | --- | --- | --- |
| Variable | β (95% CI) | p |  | β (95% CI) | p |
| Meteorological factors  (per 1 unit increase) |  |  |  |  |  |
| Temperature | - 0.005 (- 0.007 to - 0.003) | < 0.001 |  | - 0.005 (- 0.008 to - 0.002) | 0.005 |
| Diurnal temperature range | 0.025 (0.012 to 0.038) | < 0.001 |  | 0.025 (- 0.003 to 0.052) | 0.074 |
| Insolation | 0.001 (0.000 to 0.001) | 0.046 |  | - 0.00037 (- 0.001 to 0.001) | 0.418 |
| Pollutants  (per 1 unit increase) |  |  |  |  |  |
| PM_10_ | 0.003 (0.001 to 0.005) | 0.002 |  | - 0.00032 (- 0.003 to 0.002) | 0.815 |
| NO_2_ | 0.009 (0.005 to 0.012) | < 0.001 |  | - 0.001 (- 0.008 to 0.005) | 0.729 |
| SO_2_ | 0.025 (0.004 to 0.045) | 0.019 |  | - 0.004 (- 0.030 to 0.022) | 0.762 |

CI, confidence interval; SAH: subarachnoid hemorrhage
