# Supplementary material for: Monthly variations in aneurysmal subarachnoid hemorrhage incidence and mortality: Correlation with weather and pollution
Source: PLoS One. 2017 Oct 26;12(10):e0186973. doi: 10.1371/journal.pone.0186973 (PMC5658131; doi:10.1371/journal.pone.0186973)
Supplement: S4 Table — (DOCX) [file pone.0186973.s007.docx]

|  | Men | |  | Women | |
| --- | --- | --- | --- | --- | --- |
| Variable | RR (95% CI) | p |  | RR (95% CI) | p |
| Meteorological factors  (per 1 unit increase) |  |  |  |  |  |
| Temperature | 0.998 (0.994 to 1.002) | 0.289 |  | 0.993 (0.990 to 0.997) | < 0.001 |
| Diurnal temperature range | 1.049 (1.014 to 1.085) | 0.006 |  | 1.010 (0.984 to 1.036) | 0.459 |
| Insolation | 0.999 (0.998 to 1.000) | 0.081 |  | 1.000 (0.999 to 1.001) | 0.893 |
| Pollutants  (per 1 unit increase) |  |  |  |  |  |
| PM_10_ | 1.000 (0.997 to 1.003) | 0.966 |  | 1.000 (0.997 to 1.002) | 0.741 |
| NO_2_ | 1.000 (0.992 to 1.008) | 0.932 |  | 0.999 (0.993 to 1.005) | 0.639 |
| SO_2_ | 1.011 (0.978 to 1.045) | 0.515 |  | 0.986 (0.961 to 1.011) | 0.270 |
|  | < 65 years | |  | ≥ 65 years | |
| Variable | RR (95% CI) | *p* |  | RR (95% CI) | *p* |
| Meteorological factors  (per 1 unit increase) |  |  |  |  |  |
| Temperature | 0.996 (0.993 to 0.998) | 0.003 |  | 0.993 (0.988 to 0.998) | 0.005 |
| Diurnal temperature range | 1.022 (0.997 to 1.046) | 0.080 |  | 1.027 (0.989 to 1.068) | 0.167 |
| Insolation | 0.999 (0.999 to 1.000) | 0.202 |  | 1.000 (0.999 to 1.002) | 0.751 |
| Pollutants  (per 1 unit increase) |  |  |  |  |  |
| PM_10_ | 1.000 (0.998 to 1.003) | 0.889 |  | 0.999 (0.995 to 1.003) | 0.552 |
| NO_2_ | 0.998 (0.992 to 1.004) | 0.476 |  | 1.002 (0.993 to 1.010) | 0.732 |
| SO_2_ | 1.005 (0.982 to 1.029) | 0.655 |  | 0.966 (0.928 to 1.005) | 0.086 |

RR, relative risk; CI, confidence interval; PM_10_, particulate matter less than 10 mm in aerodynamic diameter; NO_2_, nitrogen dioxide; SO_2_, sulfur dioxide; SAH: subarachnoid hemorrhage
