# Supplementary material for: Monthly variations in aneurysmal subarachnoid hemorrhage incidence and mortality: Correlation with weather and pollution
Source: PLoS One. 2017 Oct 26;12(10):e0186973. doi: 10.1371/journal.pone.0186973 (PMC5658131; doi:10.1371/journal.pone.0186973)
Supplement: S5 Table — (DOCX) [file pone.0186973.s008.docx]

|  | Temperature (°C) | Atmospheric pressure  (hPa) | Precipitation (mm) |
| --- | --- | --- | --- |
| Temperature (°C) | 1 | - 0.914** | 0.711** |
| Atmospheric pressure  (hPa) |  | 1 | -0.702** |
| Precipitation (mm) |  |  | 1 |

**p <0.001
